# A mouse model to study persistent fatigue

**DOI:** 10.21203/rs.3.rs-9335120/v1

**Published:** 2026-04-30

**Authors:** Adam J. Janowski, Kazuhiro Hayashi, Ashley N. Plumb, Joseph B. Lesnak, Angela F. Smith, Lynn Rasmussen, Elizabeth Gross, Andrew A. Post, Giovanni Berardi, Marguerita E. Klein, Reid Brown, Heath Vignes, Eric B. Taylor, Andrea G. Nackley, Kathleen Sluka

**Affiliations:** University of Iowa; University of Iowa; University of Iowa; University of Iowa; University of Iowa; University of Iowa; University of Iowa; University of Iowa; University of Iowa; Duke University; University of Iowa; University of Iowa; University of Iowa; Duke University; University of Iowa

**Keywords:** Fatigue, immune system, running wheel, metabolomics, open field

## Abstract

Fatigue is characterized as a feeling of exhaustion or lack of energy, is a common symptom of multiple chronic illnesses and interferes with daily activities and quality of life. There is a limited availability of animal models to examine potential underlying mechanisms ultimately limiting development of potential therapeutic strategies. The primary purpose of this study was to develop a mouse model of persistent fatigue. A secondary purpose was to characterize potential measures of “fatigue-like” behaviors. An exploratory goal was to examine for immune and metabolic changes in the model. We examined voluntary wheel running and open field as measures of physical fatigue, and muscle and paw sensitivity as measures of pain. We also examined immune cell phenotype and plasma metabolite profiles after development of persistent fatigue. Acute stress paired with LPS or saline reduced wheel running compared to LPS alone or stress alone. Animals that received acute stress and LPS, showed a decreased ratio of T-helper to T-cytotoxic cells and reduced fatty acid metabolites 10 days after induction; there were no changes in the other groups. Thus, we characterized two unique methods, each requiring multiple stressors, to induce long-lasting fatigue-like behaviors that were associated with different mechanistic changes.

## Introduction

Fatigue is characterized as a feeling of exhaustion or lack of energy that interferes with daily activities and quality of life^1–4^. Although fatigue is recognized as multidimensional behavior, it is commonly described as a decline in physical activity^5,6^. Persistent fatigue is a common symptom of multiple chronic illnesses with prevalence rates as high as 80%^7,8^ and includes a wide range of conditions – autoimmune disorders, fibromyalgia and myalgic encephalomyelitis/chronic fatigue syndrome (ME/CFS), neurological conditions, sickness, and stress^9–12^. While fatigue can be a protective response to allow recovery from an illness, when chronic or in the absence of sickness it becomes non-protective^13–15^. Despite being common among multiple conditions, we have a limited understanding of fatigue mechanisms and few treatments.

Understanding mechanisms of persistent fatigue could lead to novel therapeutics and management strategies to address fatigue associated with chronic illnesses. Current animal models of fatigue are induced by forced physical activity such as swimming or treadmill running, immune activators such as lipopolysaccharide or poly I:C, or psychological stress models^16–21^. These existing animal models produce changes in behavioral outcomes that suggest the presence of fatigue including reductions in distance travel in open field and running wheel distance. Immune activators like LPS produce decreases in locomotor activity within 24–48h depending on dose. Forced exercise tasks, on the other hand, are likely a result of physiological muscle fatigue and may not represent persistent fatigue that occurs in chronic illnesses. While it is clear that immune activators can induce fatigue-like behaviors, those with chronic diseases often report multiple stressors^22^, each of which could lead to fatigue. In fact, multiple studies show that those who develop chronic fatigue likely had an illness prior to development, as well as a life stressor over the prior year^23–25^. Thus, animal models that combine multiple stressors may more closely mimic the clinical condition.

The immune system plays a key role in development of fatigue. The inflammatory cytokine, IL-1β, has been associated with clinical-fatigue symptoms in cancer^26,27^, IL-1β injection in mice reduces motor activity, and there is a general elevation in inflammatory cytokines in those with persistent fatigue^28,29^. Further, in individuals with fibromyalgia and ME/CFS there are alterations in lipids and energy metabolism in plasma^30–33^. Thus, stress and immune activation could lead to alterations in the immune system and metabolome and may contribute to persistent fatigue.

The purpose of this study was to develop an animal model of fatigue-like behaviors by combining an immune activator with a psychological stressor. We initially examined effects on several fatigue measures in response to an immune activator alone to confirm appropriate dosing of the immune activator and outcome measures. We then examined the combination of an immune activator with stress on fatigue-like behaviors. An exploratory aim was to examine potential systemic immune and metabolomic mechanisms involved in fatigue-like behaviors. We hypothesized that the combination of acute stress and immune stimulation would cause a reduction in activity, as a proxy for fatigue, a proinflammatory immune phenotype, and an altered metabolomic profile.

## Methods

### Animals

A total of 163 male and 163 female C57BL/6J mice (8–12 weeks old) were used. Animals were individually housed, were on a 12-hour light-dark cycle and had access to food (Inotiv Tekla, Cat. #7913; West Lafayette, IN, USA) and water ad libitum. Data were collected over several years (2020–2024), and in multiple groups. Groups were evenly distributed across days with animals randomized to groups using a random number generator. All behavioral experiments, and analysis of blood samples were done with the experimenter blinded to group. Experiments were approved by the University of Iowa Institutional Animal Care and Use Committee.

### Drugs

Drug doses were based on prior literature and preliminary experiments. Drugs were dissolved in isotonic saline at the following doses: Lipopolysaccharide (LPS) (Chondrex LPS from E.coli 0111:B4, Cat. #9028; Woodinville, WA, USA) 0.01, 0.1, 0.3, or 1.0 mg/kg, i.p.; Poly I:C (Sigma-Aldrich, Cat. #P9582; St. Louis, MO, USA) 6 mg/kg, i.p.; TAK-242 (Selleck Chemical, Cat. #S7455; Houston, TX, USA) 10 mg/kg, i.p.; morphine (Hikma, Cat. #6127; Berkeley Heights, NJ, USA) 3 mg/kg, i.p.; Modafinil (Sigma-Aldrich, Cat. #6940; St. Louis, MO, USA) 50 mg/kg, i.p.. Vehicle injections were isotonic saline.

### Restraint Stress

Animals were placed in a 50 ml Falcon tube modified to prevent escape while allowing for ample air. Size was adjustable to allow males slightly greater space than females to normalize the intensity of restraint between sexes. Animals were exposed to restraint stress for 6 hours similar to prior studies^34,35^. At the 2-hour and 4-hour timepoints animals were removed from restraint and allowed 5 minutes to walk freely in their home cage.

### Assays

#### Grip Strength

Grip strength was assessed as previously described^36^. Mice were suspended by the tail above mesh wiring connected to a force sensor. They were allowed to secure a grip on the wire mesh with their front paws or hind paws and were then pulled away until their grip broke. The average of 3 trials was calculated for the forelimb and hindlimb.

#### Fatiguing Muscle Contractions Using Electrical Stimulation:

Muscle fatigue was measured as previously described^37^. Briefly, needle electrodes were inserted into the left gastrocnemius muscle parallel to muscle fibers while the mice were anesthetized with 2–4% isoflurane. The left hindpaw was secured to a 3-D printed force plate to measure isometric gastrocnemius contractions. Maximum force production was assessed before and after fatiguing stimuli with three, 100Hz stimulus trains at 7V. Submaximal contractions were delivered with a duty cycle of 47% (3.75 seconds on, 4.25 seconds off) using trains of stimulations at 40 Hz and an amplitude of 7V for 6 minutes. Data was collected using LabVIEW software (National Instruments, Austin, TX, USA) and analyzed using a custom python script. Maximal force was determined as the average of the 3 maximum peaks before and after fatiguing contractions.

#### Voluntary Wheel Running

Mice were individually housed with a running wheel placed in each cage (Columbus Instruments, Columbus OH)^38,39^ and the number of revolutions recorded electronically. Because mice run primarily during the night cycle^40^, only the 12-hour night cycle period was used in analysis. Data were analyzed with a custom python script which calculated total distance run per day. Animals were acclimated to the running wheel for 4 days after which wheels were removed from their cages for 7 days. Running wheels were then returned to their cages. Baseline running distance was determined by taking the average of total distance run on the first night wheels were returned to cages and the 3rd and 4th acclimation days. Experimental interventions were initiated the day after wheels were returned to cages. Average wheel running distances for males (mean 3.073 km/day, SD 1.352, range 2.664–19.513 km/day) and were lower than females (mean 7.629 km; SD 2.607, range 5.919–50.871 km/day). Because of differences in baseline between animals and sexes, changes in distance run were calculated as a percent change from baseline running distance. This allowed us to examine for patterns of change in wheel running behavior.

#### Open Field

Open field testing was performed using 16-inch X 16-inch open-topped boxes as previously described^41^. The walls of each box were translucent and solid barriers placed between cages so mice could not see each other. The investigator was not present in the room during testing. Testing was performed between 10 AM and 3 PM and lumen level was 140 lux. Activity was recorded over a 30-minute period using an overhead camera (Panasonic WV-BP334) and analyzed with Limelight tracking software (Version 2.7). The testing period was evaluated using Limelight tracking and a custom python script to determine distance run, average speed, time standing still, and percent time in center of cage.

### Mechanical Withdrawal Thresholds of the Paw

Cutaneous sensitivity was tested as previously described. Specifically, mice^42^ were placed on a wire mesh platform inside clear Lucite cubicles to acclimate for 30 min, 2x per day for 2 days to the platform. Mice were tested in sets of 8, with each intervention group equally represented in each testing set with tester blinded to group and platform location. Testing was performed at the same time each day. Paw withdrawal responses were tested using a 00.4 g Von Frey filament applied to the plantar surface of the hind paw. The average response to 10 trials was calculated for both paws. An increase in the number of withdrawals was interpreted as paw hyperalgesia.

### Muscle Withdrawal Thresholds

Thirty minutes after von Frey filament testing, muscle withdrawal thresholds (MWTs) were assessed^36^. Mice were acclimated to the assay as follows. On first day they were placed in a gardener’s glove for 4 minutes 1x, followed by a second acclimation for 4 minutes. On the second day mice were acclimated for 3 minutes to the glove, 2x. During the acclimatation the hindlimb was gently manipulated On test day, mice were restrained in a gardener’s glove and the gastrocnemius muscle squeezed with force sensitive tweezers until limb withdrawal or vocalization. Three trials were performed, with 10 minute rest between trials, which were then averaged. A decrease in MWT indicates muscle hyperalgesia.

### Blood Collection

Animals were euthanized with CO_2_. Blood was drawn via cardiac puncture and immediately transferred to EDTA tubes. For mesoplex and metabolomics analyses, immediately after cardiac puncture, blood was centrifuged at 2500 rpm at room temperature for 10 minutes allowing separation of plasma from blood cells. Plasma was removed, aliquoted into 0.5 mL tubes, and stored at −80°C until analysis. For flow cytometry, whole blood was suspended in 5 ml ACK lysis buffer (GIBCO, Cat. #A10492–01; Grand Island, NY, USA), incubated for 5 minutes, centrifuged at 300 x g, twice, and washed in 5 ml DPBS (Dulbecco’s Phosphate Buffered Saline; GIBCO, Cat. #141990–144; Grand Island, NY, USA).

### Measurement of Immune System

#### MesoPlex

Cytokine levels were determined using Mesoscale Discover (MSD) assays including the V-PLEX Proinflammatory Panel 1 (K152A0H-1) and the U-PLEX TGF-β1 Panel (K152XWK-1) following the manufacturer’s instructions. Samples with a cytokine coefficient of variation greater than 20% were removed from analysis (1 IL-1β, 1 IL-2, 1 IL-5, and 6 TGF-β1). IL-4 was not analyzed since concentrations were below the measurable threshold for most samples.

##### **Flow Cytometry**:

Samples were run for flow cytometry used previously published procedures^43^. Samples were placed in a 96-well plate, treated with a viability dye (BioLegend Zombie, Cat. #UV 423107; San Diego, CA, USA) in DPBS and washed with flow cytometry staining buffer (Invitrogen eBioscience, Cat. #00-4222-26; Carlsbad, CA, USA). Fc receptor blocker (BioLegend TruStain FcX PLUS, Cat. #156603; San Diego, CA, USA) was added and samples incubated on ice for 10 minutes. Flow cytometry antibodies (Supplementary Table1) were added, determined by prior antibody titrations, and allowed to incubate for 30 minutes in the dark on ice. Samples were then washed using flow cytometry staining buffer and fixed using a 4% paraformaldehyde solution (BioLegend Fixation Buffer, Cat. #420801; San Diego, CA, USA). Samples underwent a final wash and were resuspended in flow cytometry staining buffer with an additional tandem dye stabilizer (BioLegend Tandem Stabilizer, Cat. #421802; San Diego, CA, USA). Samples were then stored in the dark at 4°C overnight. The next day, samples were analyzed on a Cytek Aurora spectral cytometer (5 laser: 355 nm, 405 nm, 488 nm, 561 nm, 640 nm). Data was processed using Cytek SpectroFlo v3.0 and data was analyzed using FlowJo v10.1. All cell populations were analyzed as percent of parent cell population.

### Metabolomics

Metabolomics analysis was run at the University of Iowa Core Facility using a broad metabolomic profiling approach that surveyed metabolites in glycolysis, pentose phosphate pathway, citric acid cycle, amino acids fatty acids, and neurotransmitters. Analysis, outlined below, has been previously published^44^.

Plasma samples were diluted in 18 volumes of ice-cold methanol/acetonitrile/water mixture (2:2:1 ratio). The mixture contained internal standards (D4-citric acid, D4-succinic acid, D8-valine, and U13C-labeled glutamine, glutamic acid, lysine, methionine, serine, and tryptophan; Cambridge Isotope Laboratories). The 1-part water was composed of sample volume and water. Plasma extraction mixtures were vortexed (10 min, RT), rotated (1h, −20°C), and centrifuges (10 min, 21,000 x g). 150 μl of the metabolite extracts were transferred to autosampler vials and dried using a SpeedVac vacuum concentrator (Thermo Fisher Scientific, Waltham, MA, USA). Dried extracts were reconstituted (30 μl of 11.4 mg/ml methoxyamine in anhydrous pyridine), vortexed (5 min), and heated (1 h, 60°C). 20 μl of N,O-Bis(trimethylsilyl)trifluoroacetamide (TMS) was then added to each sample, followed vortexing (1 min) and heated (30 min, 60°C).

Derivatized samples were analyzed by GC-MS using a TraceGold TG-5SilMS column (Thermo Fisher Scientific, Waltham, MA, USA)^45^. Derivatized samples (1μL) were injected into a gas chromatograph (Trace 1300 GC, Thermo Fisher Scientific, Waltham, MA, USA) using the following conditions: split ratio = 20:1, split flow = 24 μl/minute, purge flow = 5 ml/minute, carrier mode = Constant Flow, and carrier flow rate = 1.2 ml/minute. The GC oven temperature gradient was as follows: 80°C for 3 minutes, increasing at a rate of 20°C/minute to 280°C, and holding at a temperature at 280°C for 8 minutes. Ion detection was performed by an ISQ 7000 mass spectrometer (Thermo Fisher Scientific, Waltham, MA, USA) operated from 3.90 to 21.00 minutes in EI mode (−70eV) using select ion monitoring (SIM).

Raw data were analyzed using TraceFinder 5.1 (Thermo Fisher Scientific, Waltham, MA, USA). Metabolite identification and annotation required at least two ions (target + confirming) and a unique retention time that corresponded to the ions and retention time of a reference standard previously determined in-house. A pooled sample generated prior to derivatization was analyzed at the beginning, at a set interval during, and the end the analytical run to correct peak intensities using the NOREVA tool (Li et al., 2017) and then normalized to the summed metabolite signals to control for extraction, derivatization, and/or loading effects.

### Statistical Analysis

All data were analyzed using R Statistical Software (v4.1.2)(R Core Team, 2024) and tested for normality using Shapiro-Wilk tests. T-tests were utilized to compare two groups, one way analysis of variance (ANOVA) was used to test between groups for data with a single time point, and repeated measures (RM) ANOVA to test multiple groups over multiple timepoints. If global testing was significant, specific between group relationships were explored using (1) a Tukey’s Honest Significant Difference (HSD) test for general analysis or (2) individual t-tests for more targeted analyses to which a Bonferroni correction was applied to the p-value threshold. For Experiment 3, voluntary wheel running was separated into two time periods based on the pattern of wheel running behavior. The early phase period (Days 1–6) encompassed a time when the animals who received acute stress and LPS recovered to levels observed in the no intervention group. The late phase period (Days 7–11) encompassed the time after the animals who received acute stress and LPS had recovered. Finally, a separate planned comparison was performed between animals who received acute stress and LPS and those who received LPS alone testing for differences within the first 4 days after injection determined by pilot data.

For metabolomics, all 122 identified metabolites underwent between group analysis using a one-way ANOVA. Metabolites with p-values < 0.1 were grouped by shared chemical class or biological function and analyzed by fold-change relative to the control group. Linear mixed effects models controlling for sex and metabolite (within animal) were performed with random intercepts, but not random slopes. One-way ANOVAs followed by Tukey’s HSD tests analyzed immune cell phenotypes and cytokines. Main effects and interactions of sex were assessed for all behavior experiments. If sex effects were not significant, males and females were combined in the in the final analysis. For exploratory analysis of immune and metabolomic data, sex differences were not examined since we were not adequately powered. Power was assessed by performing initial pilot experiments to determine effect size and then sample size was determined to achieve a power of 0.80.

### Experimental Design and Analysis

#### Experiment 1: Effects of LPS on Activity Assays

To determine appropriate measures of “fatigue-like” behavior, we tested effects of the immune activator LPS (1 mg/kg, i.p.) on four activity assays. Grip strength measured maximal muscle force and was assessed before, and 24 hours after LPS in 14 mice (7M, 7F). Electrical muscle stimulation was selected as a measure of local muscle fatigue and was assessed before and 24 hours after LPS in 18 mice (9M, 9F). Voluntary wheel running measured self-selected activity and was assessed for 4 days after LPS (3M, 4F). Open field measured exploratory behavior and was assessed 24 hours after LPS in the same mice tested for running wheel activity (3M, 4F).

#### Experiment 2: Characterization of LPS effects

Since voluntary wheel running and open field testing showed reduced activity in response to 1.0 mg/kg LPS, a dose response analysis was performed (28M, 28F) for LPS (0.01, 0.1, 0.3, 1.0 mg/kg, i.p.) and compared to vehicle to select a dose of LPS to include in the model. We also tested the effects of a second immune activator Poly I:C (6 mg/kg)(8M,8F), a TLR3 agonist^46^ and compared to vehicle for comparison. We then examined if blockade of the LPS receptor TLR4 (TAK-242, 10 mg/kg, i.p.)(11M, 11F), compared to vehicle prevented decreases in voluntary wheel running induced by LPS injection (0.1mg/kg, IP). Lastly, we tested if the stimulant modafinil (50mg/kg, i.p.)(6M, 6F), compared to vehicle, prevented decreases in voluntary wheel running induced by LPS injection (0.1mg/kg, i.p.).

We also examined if LPS-induced decreases in activity were related to pain. We examined effects of systemic morphine (3 mg/kg, IP)(8M/8F) compared to vehicle after receiving LPS (1 mg/kg, IP). Morphine was given just prior to returning to animal care to assess wheel running, and morphine was given 30 minutes prior to starting the test at 24h after LPS. MWT was tested in a subset of LPS dose response experiment (0.1, 0.3, 1.0 mg/kg, i.p.)(24M, 24F).

#### Experiment 3: Characterization of the effects of stress combined with immune activation on activity measures

As both stress and acute immune activation are considered triggers for development of ME/CFS, we combined these stimuli and evaluated their impact on voluntary wheel running and open field. We initially evaluated protocols for pairing chronic versus acute stress paired with LPS using voluntary wheel running. Chronic restraint stress (2 hours/day, 5 days/week, for 3 weeks) was paired with a 1.0 mg/kg LPS the day after cessation of stress. Voluntary wheel running was recorded before, and for up to 12 days after injection. Acute restraint stress (6 hours) was paired with 0.1 mg/kg LPS and running wheels were recorded for up to 12 days after injection.

The largest and most reproducible effects in pilot data occurred when pairing acute stress with 0.1 mg/kg LPS. Therefore, acute stress and LPS were utilized to examine impact on activity measures (51M, 50F); 1 male mouse was excluded because he did not use running wheels during acclimation. The following primary groups were compared: (1) acute stress and 0.1 mg/kg LPS (7M, 8F), (2) acute stress and saline (7M, 10F), (3) acute stress and saline with rest (running wheels removed for 24h after saline injection)(8M, 7F) and (4) control (no intervention)(6M, 6F). The acute stress and saline and rest group was since clinically chronic fatigue is managed with bouts of rest during acute flares^47–52^. Additional comparison groups included (5) LPS only (8M, 7F), (6) acute stress only (5M, 4F), (7) acute stress + lower dose LPS (0.01 mg/kg, i.p.)(8M, 8F). We did a replication group of animals to compare animals receiving acute stress and LPS (9M, 10F) and compared to LPS alone (10M/9F). Another replication experiment was added to test open field on Day 10 (16M, 16F) for the acute stress + LPS, acute stress and saline, acute stress and saline and rest, and control groups. Replication experiments all analyzed wheel running and thus wheel running data were combined from the primary and replication experiments.

#### Experiment 4: Characterization of the effects of stress combined with immune activation on pain behaviors

To assess if mice developed pain after model induction, we tested mechanical sensitivity of the paw and the muscle in 32 mice (16M, 16F) as follows: (1) acute stress and LPS (4M, 4F), (2) acute stress and saline (4M, 4F), (3) acute stress and saline with rest (4M, 4F) were compared to (4) control (no intervention)(4M, 4F). Mechanical paw sensitivity and MWTs were assessed before and 24h, 72h, and 10 days after injection. Von Frey and MWT data were analyzed using the AUC of the change scores from baseline with a one-way RM ANOVA followed by a Tukey’s HSD test.

#### Experiment 5. Characterization of the immune system and metabolome in animal models of persistent fatigue

Because there is substantial evidence that alterations in the immune system and metabolome play a role in persistent fatigue^26–29,31,32^, we assessed changes in immune cell phenotype and plasma cytokine and metabolite levels. We examined the following groups: (1) acute stress and LPS (4M, 4F), (2) acute stress and saline (4M, 4F), (3) acute stress and saline with rest (4M, 4F) and (4) control (no intervention)(4M, 4F). Samples were collected 10 days after injection since this timepoint occurred during late phase activity reduction in wheel running in the acute stress and saline group.

## Results

### Experiment 1: Effects of LPS on Activity Assays

**Figure 1:**
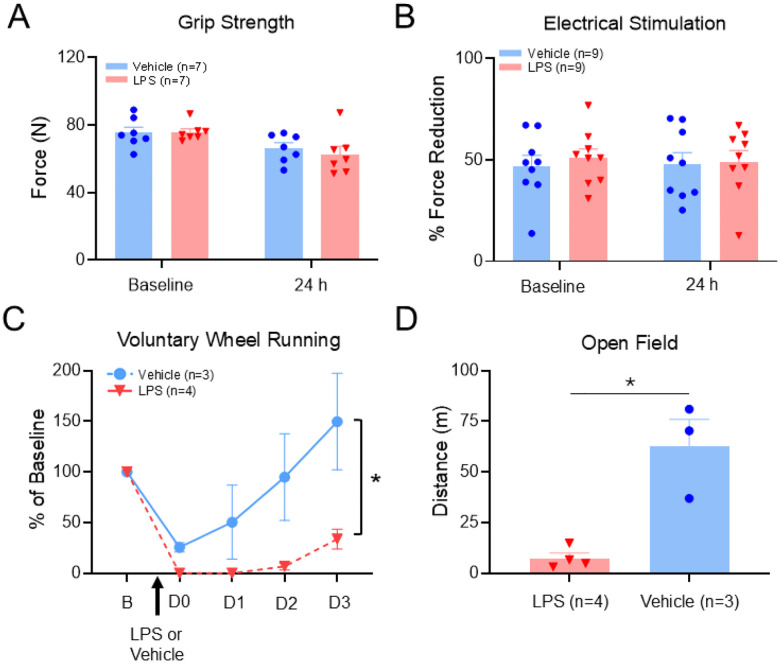
Impact of LPS on activity measures Results from behavior testing for fatigue-like behaviors before and after injection of LPS (1 mg/kg, i.p.) when compared to vehicle. There were no significant differences in grip strength (A) or electrically induced fatigue (B) 24 hours after injection of LPS when compared to vehicle. Voluntary wheel running (C) and distance traveled in the open field test (D) were significantly reduced following LPS when compared to vehicle. Data are represented as the mean ± S.E.M. *, p<0.05.

To examine effects of LPS (1mg/kg, i.p.) on “fatigue-like” behaviors, we examined the effects on 4 different potential assays. There were no changes in maximum force output (grip test) or electrically induced peripheral muscle fatigue 24 hours after LPS injection when compared to baseline values or to saline controls (*Grip Force*, group: F_1,12_=0.26, p = 0.62; time: F_1,12_=9.38, p = 0.009; group*time: F_1,12_=0.31, p = 0.59 (Fig.1a); *Peripheral Muscle Fatigue*, group: F_1,16_=0.21, p = 0.65; time: F_1,16_=0.007, p = 0.93; group*time: F_1,16_=0.086, p = 0.77 (Fig.1b)). In contrast, LPS significantly decreased voluntary wheel running activity (group: F_1,5_=6.78, p = 0.04; time: F_3,15_=11.88, p < 0.001; group*time: F_3,15_=4.53, p = 0.019) (Fig.1c) and similarly, reduced distance travelled in the open field (t = 4.8, p = 0.005)(Fig.1d). These results suggest that measures of maximal muscle force do not adequately represent systemic fatigue induced by LPS. Meanwhile, voluntary wheel running and open field activity were more sensitive measures showing decreases in wheel running and open field as previously described^13,14,53^.

### Experiment 2: Characterization of activity assays

**Figure 2:**
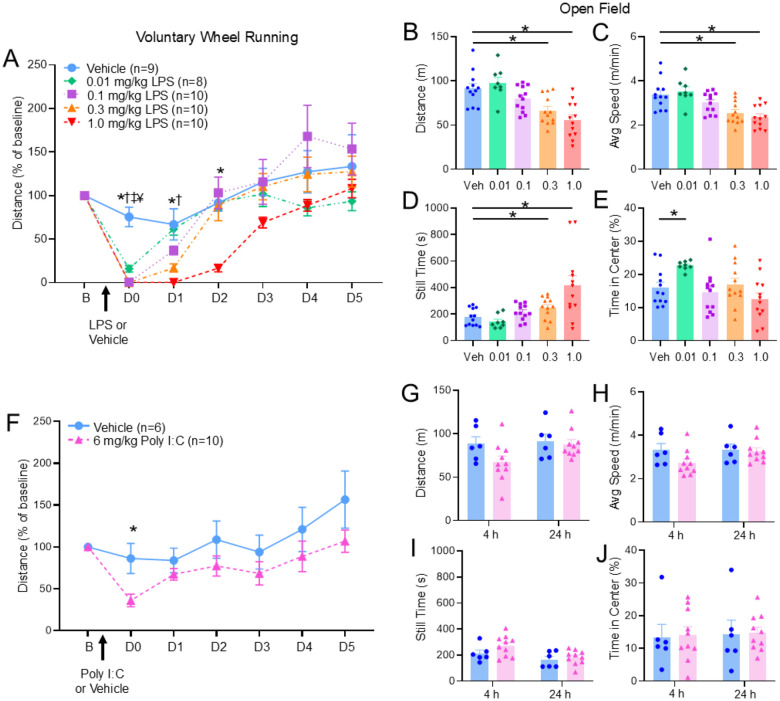
Effect of LPS and Poly I:C on voluntary wheel running and open field testing Results from wheel running and open field testing following a single injection of LPS (0.01–1.0 mg/kg, i.p.) compared to vehicle. There was a dose-dependent reduction in voluntary wheel running (A) and open field distance travelled (B), average speed (C), still time (D), and percent time in center (E). For comparison, a single dose of Poly I:C produced a shorter duration in wheel running distance (F). No changes were observed after injection of Poly 1:C in the open field test for distance travelled (G), average speed (H), still time (I), and percent time in center (J). Data are represented as the mean ± S.E.M. Significance (p<0.01) is represented as ¥ for 0.01, † for 0.1, ‡ for 0.3, and * for 1.0; p<0.01 for LPS doses, and *p<0.0.05 for Poly I:C.

To determine an appropriate dose for LPS in subsequent experiments, we examined the impact of several different doses of LPS on wheel running and open field behavior. LPS induced a dose-dependent decrease in voluntary wheel running activity over a 6-day period (group: F_4,41_=3.51, p = 0.015; time: F_5,205_=86.4, p < 0.001; group*time: F_20,205_=3.77, p < 0.001)(Fig.2a). When compared to vehicle, there were decreases in distance run in all groups injected with LPS on the day of injection (Vehicle: p < 0.001, δ = 2.96; 0.1 mg/kg: p < 0.001, δ = 4.13; 0.3 mg/kg: p < 0.001, δ = 4.16; 1.0 mg/kg: p < 0.001, δ = 4.17) (Fig.2a). These reductions in running remained significant for the 0.3 and 1.0 mg/kg LPS groups at Day 1 after injection (0.3 mg/kg: p = 0.02, δ = 1.52; 1.0 mg/kg: p = 0.006, δ = 2.06) and the 1.0 mg/kg group at Day 2 after injection (p < 0.001, δ = 3.64)(Fig.2a).

**Figure 3:**
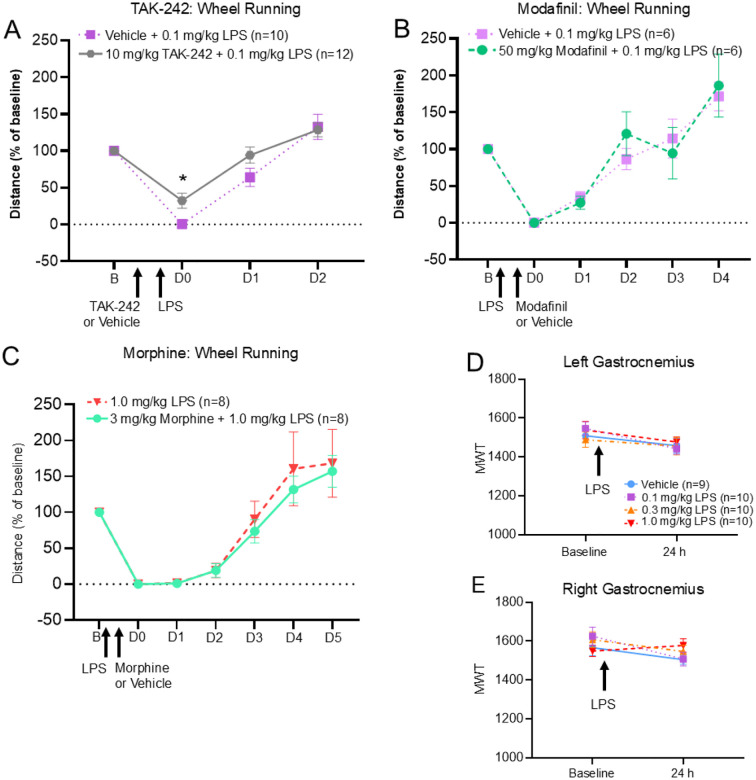
Validation experiments Voluntary wheel running results in animals injected (A) TAK-242 (10 mg/kg, i.p.) injected 10 minutes prior to LPS, (B) Modafinil injected (50mg/kg, i.p.) 4 hours after LPS, (C) morphine (3mg/kg, i.p.) injected 4 hours after LPS. Each drug was compared against a vehicle control group. Tak-242 attenuated wheel running when compared to vehicle controls. Modafinil and morphine had no effect on wheel running. (D,E) Muscle withdrawal thresholds for left and right gastrocnemius showed no changes 24 hours after systemic injection of LPS (0.1–1.0mg/kg, i.p.) when compared to vehicle. Data are represented as the mean ± S.E.M. *, p<0.05.

For open field activity, LPS-induced a dose-dependent decrease 24 hours after injection when compared to vehicle in: 1) *distance travelled* (F_4,51_=10.29, p < 0.001) for animals in the 0.3 mg/kg and 1.0 mg/kg groups (p = 0.003, δ = 1.39; p < 0.001, δ = 1.78)(Fig. 2b); 2) *average speed* (F_4,51_=8.89, p < 0.001) in the 0.3 mg/kg and 1.0 mg/kg groups (p = 0.003, δ = 1.35; p = 0.001, δ = 1.68)(Fig.2c); 3) *time standing still* (F_4,51_=7.39, p < 0.001) in the 0.3 mg/kg and 1.0 mg/kg groups (p = 0.029, δ = 0.96; p = 0.009, δ = 1.26) (Fig.2d); and 4) *percent of time in center* (F_4,51_=3.82, p = 0.009) in the 0.01 mg/kg group (p = 0.002, δ = 1.5)(Fig.2e).

To determine if comparable results were found with another immune activator, we examined the impact of the TLR3 agonist Poly I:C on activity. Poly I:C produced a reduction in wheel running on the day of injection when compared to the vehicle group (p = 0.037, δ = 1.55)(Fig.2f). Mice tested with Poly I:C showed no significant differences in open field activity 4h or 24h post injection (Fig.2g–2j).

We then examined if the effects of LPS on wheel running were the result of activation of immune mechanisms by blocking the LPS binding site (TLR4 receptors) or general loss of activity by examining effects of the stimulant Modafinil. When TLR4 antagonist TAK-242 was injected 3 hours prior to LPS, animals ran a greater distance in running wheels compared vehicle controls (group: F_1,20_=2.14, p = 0.16; time: F_2,40_=108.74, p < 0.001; group*time: F_2,40_=3.46, p = 0.04)(Fig.3a). This effect was present only on the first night of running after LPS-injected mice received either TAK-242 or vehicle (p = 0.01, δ = 0.9) (Fig.3a). Treatment with Modafinil after LPS injection caused no change on wheel running (Fig.3b). In the open field test, distance travelled, average speed, still time, and percent time in center of cage was unaffected by TAK-242 or Modafinil (Supplemental Fig.1).

To determine if decreases in voluntary wheel running were related to increased pain, we examined the effects of morphine, a mu-opioid receptor agonist, on wheel running. Morphine had no effect on reductions in voluntary wheel running or open field induced by LPS (Fig.3c, Supplemental Fig.1). We then examined if LPS altered muscle sensitivity. LPS (0.1–1.0 mg/kg) had no effect on MWT 24 hours after injection (Fig. 3d, 3e).

### Experiment 3: Characterization of the effects of stress combined with immune activation on activity

To determine if acute stress could enhance the reductions in wheel running or open field, we combined acute stress with a low dose LPS (0.1 mg/kg) and compared to LPS alone. Combining acute stress with LPS enhanced reductions in wheel running compared to LPS alone; there was no sex-specific effect (group: F_1,36_=6.2, p = 0.017; time: F_1,112_=548.2, p < 0.001; group*time: F_1,112_=12.9, p < 0.001)(Fig.4a). Area under the curve confirmed a greater reduction in wheel running when LPS was combined with acute stress compared to LPS alone (p = 0.017, δ = 0.81)(Fig.4b). Interestingly, chronic stress combined with LPS had no effect on wheel running when compared to LPS alone (Supplemental Fig.2).

**Figure 5:**
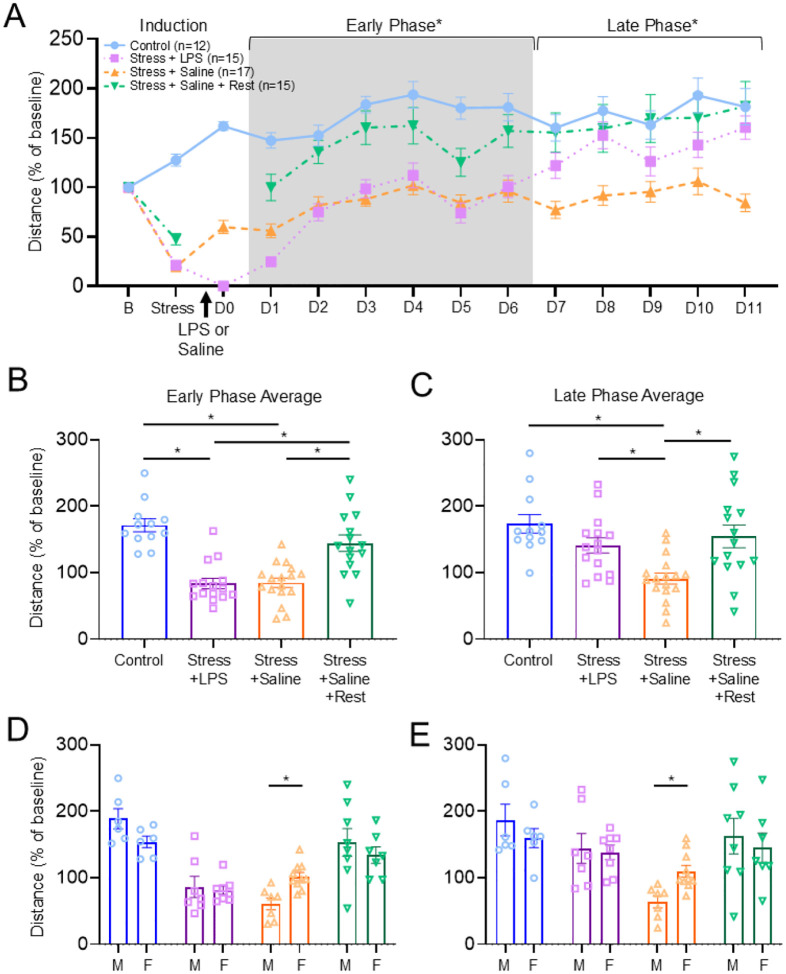
Voluntary wheel running over 12 days is reduced by acute stress combined with LPS or saline (A) Voluntary wheel running distance by day measured before and after induction of the models for the primary groups: acute stress and LPS, acute stress and saline, acute stress and saline and rest, and controls. Both acute stress and LPS and acute stress and saline produced a persistent decrease in running wheel activity compared to controls. Interesting providing a 24h period of rest at the time of LPS injection prevented the decrease in wheel running after acute stress combined with saline. Average running in the (B) early phase (Days 1–6) and (C) late phase periods (Days 7–11) after model induction of the models. Average running in the (D) early phase and (E) late phase periods after model induction separated by sex and show sex differences in the stress and saline model with males showing greater reductions in wheel running than females. Data are represented as the mean ±S.E.M. *, p<0.013.

Based on these results, we examined voluntary wheel running using 4 groups of animals over a longer 12-day period. We compared acute stress and LPS (0.1 mg/kg, i.p.) to acute stress and isotonic saline and no intervention control groups, and acute stress and saline with a 24h rest after the injection of saline. In 3 groups that received acute stress, voluntary wheel running decreased significantly in all groups relative to no intervention controls (Fig.5a). Acute stress alone and 0.1 mg/kg LPS alone showed an immediate decrease in running wheel behavior that returned to values observed in the naïve group by D1 (Supplemental Fig.3).

In the early phase (Days 1–6), there were significant differences in distance run (group: F_3,55_=23.2, p < 0.001; sex: F_1,55_=0.01, p = 0.91; group*sex: F_3,55_=2.88, p = 0.04)(Fig.5b). Specifically, mice that received acute stress and LPS ran less than naïve controls (p < 0.001, δ = 2.74) and mice that received acute stress and saline with rest (p < 0.001, δ = 1.51). Mice that received acute stress and saline ran less than naïve controls (p < 0.001, δ = 2.76) and mice that received acute stress and saline with rest (p < 0.001, δ = 1.52). Interestingly, there were sex differences in wheel running in the group that received acute stress and saline with males showing a greater reduction in wheel running than females (p = 0.003, δ = 1.91) (Fig.5c).

In the late phase (Days 7–11), there were significant group differences for wheel running (group: F_3,55_=7.37, p < 0.001; sex: F_1,55_=0.01, p = 0.93; group*sex: F_3,55_=1.75, p = 0.17)(Fig.5a,d). Specifically, mice that received acute stress and saline ran significantly less than controls (p < 0.001, δ = 2.02), acute stress and LPS (p = 0.03, δ = 1.26), acute stress and saline with rest (p = 0.003, δ = 1.22). Sex differences in the late phase were present in the stress and saline group with males showing a greater reduction than females (p = 0.003, δ = 1.74)(Fig.5e).

Open field testing on Day 5 shows significant group*time interactions for distance travelled (p = 0.03), average speed (p = 0.02), and time standing still (p = 0.009) on Day 5; however there were no differences between groups with post-hoc tests (Supplemental Fig.4). On Day 10, open field testing was not different between groups (Supplemental Fig.4).

### Experiment 4: Characterization of the effects of stress combined with immune activation on pain behaviors

**Figure 6:**
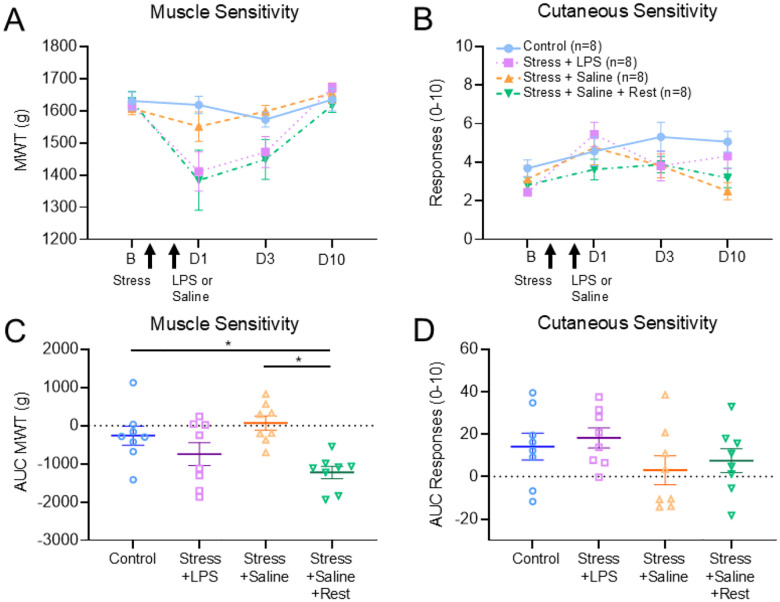
Minimal changes in pain behaviors after induction of the models Line graphs show (A) Muscle sensitivity and (B) cutaneous sensitivity before and 1(D1), 3 (D3) and 10 (D10) days after induction for the acute stress and LPS, acute stress and saline, acute stress and saline and rest, and controls. Scatter plots show AUC of the change score for (C) muscle and (D) cutaneous sensitivity. Data are represented as the mean ±S.E.M. *, p<0.013 tested relative to control group.

To determine the impact of acute stress and immune activation on pain behaviors we examined cutaneous and muscle sensitivity to mechanical stimuli before and after induction of the models (Fig.6). For changes in MWT, there were significant differences for group, sex, group*time and group*sex (group: F_3,23_=7.28, p = 0.001; sex: F_1,23_=3.53, p = 0.07; group*time: F_6,48_=2.61, p = 0.02; group*sex: F_3,23_=3.56, p = 0.02) (Fig.6a). Specifically, MWT was lower in the group that received acute stress and saline with rest compared to controls (p = 0.03, δ = 1.61)(Fig.6b) and acute stress and saline (p = 0.003, δ = 2.64) (Fig.6c). Supplemental Fig.5 shows the sex differences by group with the female mice showing decreases in the stress with saline and rest group and the male mice showing decreases in both the stress with LPS and stress with saline and rest groups. For cutaneous sensitivity, there were no differences between groups, sexes, or changes over time (Fig.6b,d).

### Experiment 5. Characterization of the Immune and Metabolomic Differences

#### Immune cell phenotype

**Figure 7:**
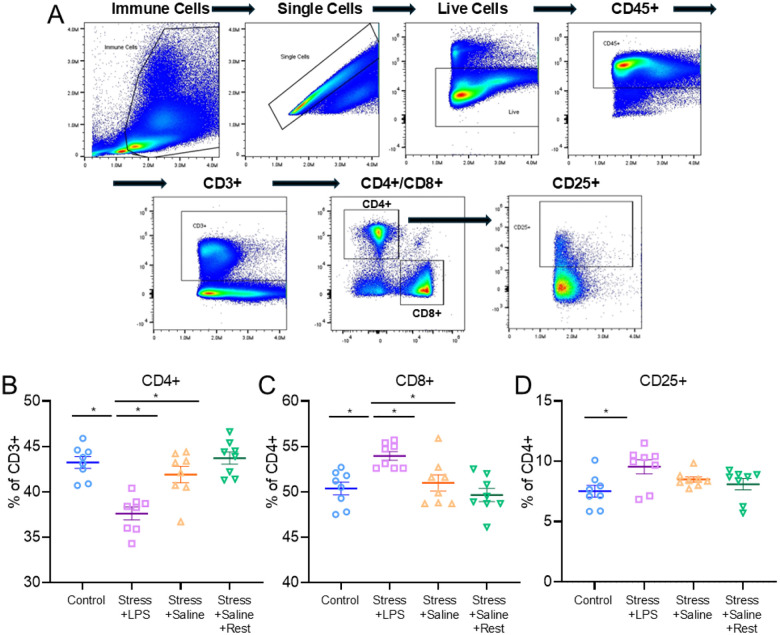
Altered phenotype of T-Cells 10 days after induction of the stress and LPS group Results from spectral flow cytometry of whole blood drawn at Day 10 after model induction. A. Gating strategy for flow. B. Percent expression of CD4+ T-cells (B) and CD8+ cytotoxic T-cells (C) and CD25+ regulatory T-cells (D) are shown in scatter plots. Data are represented as the mean ± S.E.M. *, p<0.05.

We utilized spectral flow cytometry to explore immune cell phenotypes 10 days after induction of the models as that was the timepoint with greatest late phase group differences in voluntary wheel running (Fig.7). There were significant differences in 3 cell populations: CD4 + T helper cells (F_3,24_=14.04, p < 0.001)(Fig.7b), CD8 + cytotoxic T cells (F_3,24_=7.03, p = 0.001)(Fig.7c), and CD25 + regulatory T cells (F_3,24_=3.36, p = 0.02)(Fig.7d). There were lower percentages of CD4 + cells in mice that received acute stress and LPS compared to animals who received acute stress and saline (p = 0.001, δ = 1.86), acute stress and saline with rest (p < 0.001, δ = 3.11), and naïve controls (p < 0.001, δ = 2.94)(Fig.7b). Inversely, there were higher percentages of CD8 + cells in mice who received acute stress and LPS compared to animals that received acute stress and saline (p = 0.03, δ = 1.47) acute stress and saline with rest (p = 0.001, δ = 2.46), and naïve controls (p = 0.007, δ = 2.13)(Fig.7c). There were higher percentages of CD25 + cells in mice that received acute stress and LPS compared to naïve controls (p = 0.02, δ = 1.32) (Fig.7d). There were no differences between groups in other cell populations measured including CD45+, CD3+, CD183+, CD184+, CD19+, Ly6C+ monocyte subsets, NK cells or NK cell subsets (Supplemental Fig.6). The complete gating strategy is provided in Supplemental Fig.7.

#### Plasma Cytokines

There were no differences in plasma cytokine levels between groups for IFN-γ, IL-1β, IL-2, IL-5, IL-6, IL-10, KC-GRO, TNF-α, or TGF-β1 (Supplemental Table1; Supplemental Fig.8). All cytokines were assessed for sex differences and interestingly, females demonstrated significantly higher levels of IL-5 compared to males across all groups (p < 0.001); however there was not group*sex interaction (Supplemental Table1).

#### Metabolomics

**Figure 8:**
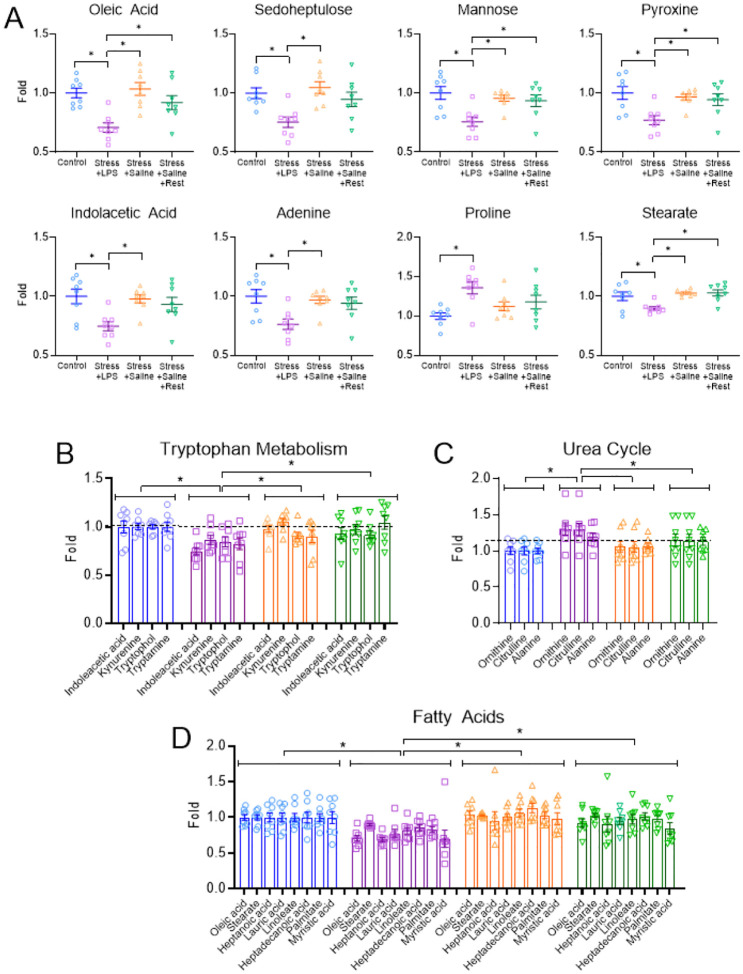
Altered metabolomics of plasma in the group treated with stress and LPS. Results from metabolomics (GC-MS) on Day 10 after model induction. Graphs show the top 8 significantly different metabolites (A), tryptophan metabolites (B), urea cycle metabolites (C), and fatty acids metabolites (D) in each group. Data are represented as the mean ±S.E.M. *, p<0.05.

Metabolomic analysis showed 32 of 122 metabolites with p ≤ 0.1, 24 with p < 0.05, and 8 with p < 0.01 (Supplemental Table2). Of the top eight metabolites, significance was driven by differences between animals that received acute stress and LPS (Fig.8a). There were no differences between the mice that received acute stress and saline or acute stress and saline with rest compared to naive controls, and there were no group*sex interactions. Metabolites that were lower in the animals that received acute stress and LPS relative to controls included oleic acid (p < 0.001, δ = 2.6), sedoheptulose (p = 0.002, δ = 1.93), mannose (p = 0.004, δ = 1.75), pyridoxine PN (p = 0.005, δ = 1.73), indoleacetic acid (p = 0.005, δ = 1.74), adenine (p = 0.006, δ = 1.66), stearate (p = 0.03, δ = 1.31), β-Hydroxybutyrate (p = 0.001, δ = 2.32), while proline (p = 0.002, δ = 2.06) was higher (Fig.8a).

We then grouped metabolites by function that demonstrated p ≤ 0.1 to examine potential biological pathways. There were differences within fatty acids (oleic acid, stearate, heptanoic acid, lauric acid, linoleate, heptadecanoic acid, palmitate, myristic acid), urea cycle metabolites (ornithine, citrulline, alanine), and tryptophan metabolites (indoleacetic acid, kynurenine, tryptophol, tryptamine). Compared to controls, tryptophan metabolites were lower in mice that received acute stress and LPS (β=−0.18, p < 0.001)(Fig.8b). Urea cycle metabolites were higher than controls for mice that received acute stress and LPS (β = 0.26, p = 0.003) (Fig.8c). Fatty acid metabolite levels were lower than controls for those that received acute stress and LPS (β=−0.21, p < 0.001)(Fig.8d).

## Discussion

We showed that voluntary wheel running and open field behavior, measures that have a volitional component, were reduced by an immune activator alone, suggesting immune activation produces fatigue-like behaviors. Using voluntary wheel running and open field testing, the current study showed that acute stress in combination with either systemic LPS or saline reduced wheel running behavior in mice for up to 2 weeks; longer effects occurred in the group receiving acute stress combined with saline. Alterations in the metabolome and immune system only occurred in the stress and LPS group, but not the stress and saline group. Thus, we examined characterized two different models that produce fatigue-like behaviors that are likely produced by different mechanisms.

### Preclinical measures of fatigue

Measuring fatigue in preclinical work is challenging given its diffuse and subjective nature. The robust decreases in voluntary wheel running in the current study in response to LPS are a proxy of fatigue, and agree with prior studies showing reduced voluntary wheel running and open field activity using single doses of LPS of varying concentration (0.83–3mg/kg)^14,20,54–56^. These results are consistent with human studies showing systemic IL-6 produces fatigue and reduced activity levels^57^ and support a role of the immune system in the generation of fatigue-like behaviors. We expanded these data and show a dose-response effect with LPS on voluntary wheel running and open field activity, partial attenuation of LPS-induced reduction in wheel running by blockade of the LPS-receptor TLR4, and similar effects in males and females. Thus, immune system activation can result in fatigue-like behaviors, a key component of sickness behavior^14,58^.

LPS had small effects on open field, and no effects in grip strength or electrically induced muscle fatigue. In mice, both open field and wheel running are innate behaviors that are self-selected, volitional activities^41,59^. Wheel running involves a longer testing time (12h wake cycle vs 30 min) and greater total activity volume and thus may be more impacted by fatiguing stimuli. On the other hand, grip force, a reflexive response that measures maximum muscle contractio^60^, and electrical stimulation of the muscle, a measure of muscle fiber fatiguability^61^, more likely represent effects at the local muscle level. Thus, LPS had no effect on peripherally driven measures of fatigue but did impact central or systemic mechanisms of fatigue.

We also show that LPS alone does not change pain behaviors and running wheel activity is not responsive to the analgesic morphine, suggesting that LPS-induced reductions in activity are not related to pain. On the other hand, prior studies show that induction of paw inflammation reduces wheel running that can be restored with the analgesic morphine, suggesting in those studies the reduced activity is related to pain^62,63^. Wheel running is sensitive to a variety of stimuli^64^ and thus should be interpreted in the context of the study.

### Multiple stressors produce longer-lasting fatigue behaviors

The current study showed acute stress combined with a low-dose LPS produced longer-lasting reductions in wheel running when compared to LPS-alone or stress-alone. These data are consistent with previous reports that stress, as a primer for LPS, can increase depressive behaviors^55^, and LPS combined with physical activity reduces wheel running^20,65^. Surprisingly, stress paired with an isotonic saline injection produced a longer reduction in voluntary wheel running than when paired with LPS. Consistent with this, prior studies show isotonic saline injection produces pronounced effects on exploratory behavioral outputs, and changes in brain mRNA^66–68^ and proteins^69–72^ across multiple sites including the brainstem, hypothalamus, amygdala, cortex, and thalamus without changes in systemic cytokines. Thus, isotonic saline injection can also have widespread effects and thus when combined with acute stress can produce an exaggerated response.

Interestingly, we show that 24h of rest on the day of saline injection prevented the late phase decrease in voluntary wheel running observed in the stress and saline group, suggesting that rest immediately after injection protected the mice from development of fatigue. Consistent with this hypothesis, athletes that maintain training regimens during the initial stages of an acute illness have been linked to the development of ME/CFS^73^, and acute exercise can enhance fatigue in individuals with ME/CFS^74–77^. Similarly, a single bout of exercise in mice can enhance pain^37^, and activate the immune system to enhance inflammatory cytokine release^78–80^. On the other hand, longer-term regular exercise enhances release of anti-inflammatory cytokines in humans and animals^81–85^.

Pain is a common symptom in multiple chronic diseases and often co-occurs with fatigue including in individuals with fibromyalgia, ME/CFS and long-COVID^8,86^. We observed increased muscle pain in the stress and saline with rest group, but not in the stress and saline mice that remained in the running wheels. This is consistent with data from our laboratory and others showing that running wheel activity is protective against development of muscle pain^38,87^. These findings are also consistent with studies of fatigue in individuals with ME/CFS that highlight the importance of rest between activity in managing their fatigue^47–52^. Together, these data suggest that in mice one day of rest protects against development of fatigue, while voluntary running wheel activity protects against development of muscle pain.

### Changes in the immune system and metabolome may contribute to persistent fatigue

The acute stress and LPS group showed alterations in immune cell proportions. Specifically, there were lower CD4+ (T-helper) cells, elevated CD8 + cells (Cytotoxic T-cells) and elevated levels of T-regulatory cells 10 days after model induction. These findings are consistent with findings from individuals with ME/CFS where several studies also show lower CD4+/CD8 + ratios^88–91^; however, not all detect this difference^90,92,93^. Lower CD4+/CD8 + ratios are thought to be related to impaired immune function^94–96^ and greater risk of infection^95,97^, while elevated T-regulatory cells are associated with immune suppression^98,99^. In the current study, despite changes in immune cell phenotypes, there were minimal changes in circulating cytokines.

Similar to immune changes, plasma metabolomic analysis demonstrated strong differences in the group that received acute stress and LPS. Along with several specific metabolites, differences were greatest throughout fatty acids, tryptophan-related, and urea cycle metabolites, which are consistent with previous research in individuals with ME/CFS^30,32,100,101^. Fatty acids are known regulators of immune homeostasis demonstrating both pro and anti-inflammatory effects on T-cells, macrophages, and neutrophils^102,103^. Depleted tryptophan metabolites may inhibit T-cell proliferation^104^ while elevated urea cycle metabolites may inhibit T-cell and macrophage proliferation^105^. There were also significantly lower levels of individual metabolites (oleic acid, sedoheptulose, mannose, pyridoxine, indoleacetic acid) which have a known anti-inflammatory influence on the immune system^106–113^ suggesting a loss of the protective effects of the immune system. Thus, LPS combined with stress produced long-lasting changes in immune cell phenotype and the metabolome, suggesting animals remain vulnerable to subsequent stimuli.

Surprisingly, there were no changes in the immune system or metabolome in the group with acute stress and saline, despite behavioral changes. Indeed, as stated above, saline can both produce changes in mRNA, proteins and peptides across the central nervous system^66–72^ Similarly, stress can produce changes in transcription factors, intracellular messengers, glial cell activation and inflammatory mediators across sites within the central nervous system^114–118^. Thus, the saline and stress model may be more representative of a centrally-maintained fatigue condition without systemic mechanisms. Future studies should confirm this hypothesis.

### Limitations

We tested acute stress paired with an immune activator but those were not the only stressful events mice were exposed to. Social isolation, transportation, handling, and behavior testing are known stressors and can affect wheel running^71,119,120^. Therefore, while restraint stress was the dominant stressor provided, smaller accessory stressors were an inherent part of experimental design and testing and could have played a role in model induction and maintenance. While we are interpreting the decrease in running wheel activity as a fatigue-like behavior, it is possible that other factors also affected the running wheel behavior. However, we did not find changes in muscle strength or peripheral muscle fatigue, suggesting the reduced activity was related to induction of the model itself. It should be noted that LPS induced sickness behavior, of which fatigue is a main component. Combining acute stress with LPS produced a longer-lasting effect that either stressor alone, and combining saline with acute stress also produced the effect. We thus interpreted the data as a fatigue-like response beyond that observed by sickness alone. Finally, immune characterization was powered to detect meaningful differences in cell phenotype but not plasma cytokines. Also, while we were powered to detect sex differences in many experiments, this was not the primary aim, and thus not all experiments were powered for sex. Future experiments can use the exploratory mechanistic data to design and adequately power to test novel hypothesis.

## Conclusions

The pairing of acute stress with systemic immune activation can produce fatigue-like behaviors with an extended impact on the immune system and metabolome. Meanwhile, acute stress paired with systemic saline injection produces longer-lasting fatigue-like behaviors without impacting the peripheral immune system or metabolome. Thus, we developed two unique models to examine underlying mechanisms of fatigue-like behaviors associated with chronic disease (Fig.9). These two models likely have differing underlying mechanisms and thus can be used to probe unique mechanisms that contribute to persistent fatigue.

## Supplementary Files

This is a list of supplementary files associated with this preprint. Click to download.

- suppelementalfiguresAdamMarchsubmission.pptx

## Data Availability

The datasets generated during and/or analyzed during the current study are available from the corresponding author on reasonable request.

## New & Noteworthy

This study characterized two unique methods for inducing persistent chronic fatigue-like behaviors in mice. Specifically, combining acute stress with either a systemic immune activator or saline led to persistent fatigue. The combination of stress with the immune activator showed systemic changes in the immune and metabolic pathways that were not found in the mice with stress combined with saline. These models may serve to examine unique underlying mechanisms of persistent fatigue in chronic illnesses.
